# Novel Y chromosome breakpoint in an infertile male with a de novo translocation t(Y;16): a case report

**DOI:** 10.1007/s10815-012-9886-8

**Published:** 2012-11-15

**Authors:** Yu-Ting Jiang, Hong-Guo Zhang, Rui-Xue Wang, Yang Yu, Zhi-Hong Zhang, Rui-Zhi Liu

**Affiliations:** Center for Reproductive Medicine, The First Bethune Hospital of Jilin University, Changchun, Jilin 130021 China

## Introduction

Cytogenetic anomalies are an important cause of male infertility. The rate of chromosomal rearrangement ranges from 10–15 % in azoospermic males [1, 2]. Y;autosome translocations can be found in both fertile and sterile males, depending on the Y chromosome breakpoint and/or the autosome involved [3, 4]. It is generally assumed that fertile males have a Y chromosome breakpoint at Yq12, the genetically inert heterochromatic block, whereas the Y chromosome breakpoint in sterile males is in the distal Yq11 euchromatic region that contains the azoospermia factor (AZF) locus [5]. To date, there have been only five cases reported of a balanced reciprocal (Y;16) translocation associated with male infertility. Here, we present molecular and cytogenetic characterization of a de novo Y;16 translocation with breakpoints at Yp11 and 16q11 in an adult azoospermic male.

## Case report

A 38-year-old male presented with primary infertility having had 6 years of regular unprotected intercourse, The patient’s medical history was unremarkable for infertility risk factors. Physical examination revealed normal penis and pubes. The left and right testicular volumes were both 15 ml. Three routine semen analyses, performed according to the World Health Organization guidelines [6], revealed no sperm. Reproductive hormone levels were normal for prolactin (315 μIU/mL; normal range 86–324 μIU/mL), luteinizing hormone (3.1 mIU/mL; normal range 1.7–8.6 mIU/mL), follicle-stimulating hormone (3.3 mIU/mL; normal range 1.5–12.4 mIU/mL), testosterone (6.4 ng/mL; normal range 2.8–8.0 ng/mL), and estradiol (29.98 pg/mL; normal range 7.63–42.6 pg/mL). Appropriate voluntary written consent was obtained from the patient and his family. This study was approved by the Chinese Association of Humanitarianism and Ethics.

## Chromosomal analysis and fluorescent in situ hybridization (FISH)

Cytogenetic investigations were performed on the patient’s chromosomes obtained from peripheral blood lymphocytes, which were cultured in RPMI Medium 1640 (GIBCO, Invitrogen Carlsbad, CA, USA), phytohemagglutinin (Shanghai Yihua Medical Technology Co., Ltd., Shanghai, China), and fetal bovine serum (Beijing Dingguo Biotechnology, Beijing, China) for 72 h, followed by treatment with 50 μg/ml colcemid. Metaphase chromosome spreads were studied by standard GTG and CBG banding procedures, which included using trypsin and Giemsa for G-banding and barium hydroxide for C-banding.

FISH was performed on 30 metaphase chromosome spreads using a mixture of probes specific for DXZ1 and DYZ3 (CSP X Spectrum green and CSP Y Spectrum red; Beijing GP Medical Technologies, Beijing, China), and a chromosome-specific probe for CBFB (GLP 16 banding at 16q22, Spectrum red; Beijing GP Medical Technologies).

## Molecular deletion analysis

Multiplex PCR amplification of nine sequence-tagged site markers was used to detect AZF region microdeletions on the Y chromosome [7]. These markers were: sY84, sY86 for AZFa; sY27, sY134, and sY143 for AZFb; sY152, sY157, sY254, and sY255 for AZFc.

## Testicular cytology

A fine-needle aspiration biopsy was performed under local anesthesia in the pole of the patient’s right testis. The retrieved specimen was washed three times in phosphate-buffered saline, spread onto glass slides, and air-dried. The specimens were then fixed in 95 % alcohol and stained with hematoxylin-eosin. The cells were examined under high magnification using a 40× light microscope and the spermatogenic status was classified according to the Meng system [8].

## Results

**Fig. 1:**
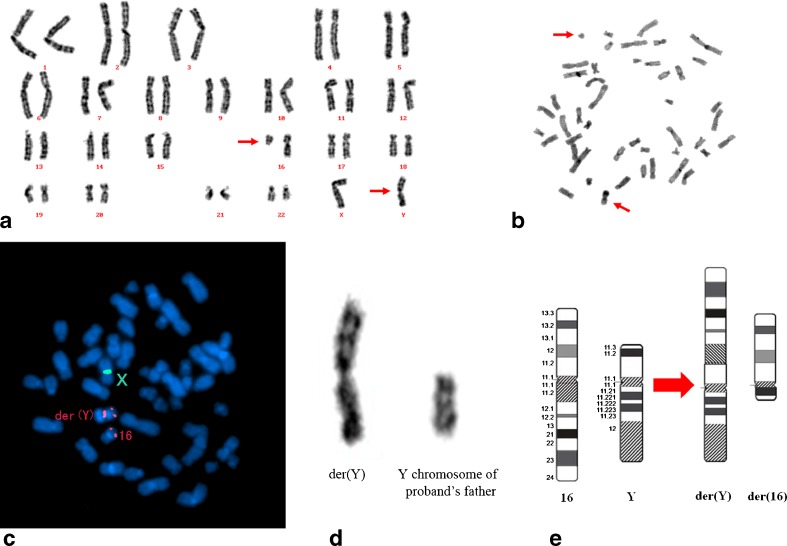
**a**, GTG; **b**, CBG. Arrows indicate the derivative chromosomes; **c**, Two-color FISH with DNA probe specific for DXZ1 (*green*), DYZ3 (*red*), CBFB (*red*), and DAPI (4′,6-diamidino-2-phenylindole; *blue*) staining; **d**, Derivative Y chromosome of proband and Y chromosome of proband’s father; **e**, Ideogram of Y;16 translocation

A G-banded karyogram of the proband revealed a balanced translocation between chromosomes Y and 16, although the exact position of the breakpoints was unclear. Initially, we assumed that the breakpoints were at Yq12 and 16p13 (Fig. 1a). A C-banded karyogram was also performed (Fig. 1b). FISH confirmed that the breakpoints were at Yp11 and 16q11 (Fig. 1c). The parents of the proband did not have any chromosomal rearrangements. However, the Y chromosome morphology of the patient’s father was similar to that of part of the patient’s derivative chromosome (Fig. 1d). Chromosome ideograms are shown in Fig. 1e.

At the molecular level, no microdeletions were detected in the AZF region of the Y chromosome in this infertile man (data not shown). Cytological analysis of a testicular biopsy specimen showed complete maturation arrest (data not shown). Neither sperm nor spermatids were detected. Sperm maturation had stopped in the early stages of spermatogenesis.

## Discussion

The frequency of Y;autosome translocations in the general population is approximately 1 in 2000 [9, 10]. Translocations between the Y and a non-acrocentric chromosome are rare and often lead to infertility [11]. The mechanisms of Y;16 translocation and associated phenotypes have been revealed by meiotic studies of the synaptic behavior of the XY-autosome quadrivalent [4, 12, 13]. Indeed, in cases with a translocation, most of the X and Y chromatin is not paired during male meiosis at the zygotene and pachytene stages [14]. At the pachytene stage, the XY bivalent may be connected with the quadrivalent. The first pachytene checkpoint is activated by this particular structure and decreased numbers of cells reach the later pachytene stages. The second breakdown of the meiotic process could be caused by inactivation of genes located in the regions associated with the XY bivalent [15, 16]. Gene inactivation would block transcription of some of these genes, which in turn could trigger an apoptotic response.

Y;16 reciprocal translocations reported in previous studies [17–22] are shown in Table 1. To our knowledge, our patient is the first case of reciprocal translocation t(Y;16) with breakpoints at Yp11 and 16q11 to be associated with male infertility. In other cases of Y;16 translocation, the breakpoints were at Yq11 or Yq12, and the phenotype was dependent on the precise breakpoint localization and the nature of the Yq material lost [23, 24]. However, molecular studies in our patient revealed no microdeletions in the AZF region. We assume that there are unknown spermatogenesis regulatory gene(s) at Yp11 whose expression is affected by this chromosomal rearrangement. Alternatively, the translocation may affect the influence of the heterochromatin region; previous studies have reported a disturbance of meiosis related to the heterochromatin region of chromosomes 1, 9, 16, and the interphase nucleolus [25, 26].

**Table 1:** Genotype–phenotype correlation in adult males with Y;16 translocation

References	Karyotype	Origin	Molecular analysis	Phenotype	
				Sperm count	Testicular histology
Faed et al.,1982 [16] ^a^	46,X,t(Y;16)(q11;q13)	de novo	NP	Oligozoospermia	Partial block at spermatid formation Scanty sperm
Abeliovich et al.,1986 [17] ^a^	46,X,t(Y;16)(q11;p13)	de novo	NP	Azoospermia	Maturation arrest of spermatogenesis
Gregor et al., 1990 [18]	46,X,t(Y;16)(q12;q11-12)	de novo	NP	Azoospermia	NP
Giltay et al., 1998 [19] ^a^	46,X,t(Y;16)(q11.21;q24)	de novo	No deletion of AZF	Oligozoospermia	NP
Gunel et al., 2008 [20]	46,X,t(Y;16)(q12;q13)	de novo	NP	Azoospermia	Maturation arrest
Present study	46,X,t(Y;16)(p11;q11)	de novo	No deletion of AZF	Azoospermia	Maturation arrest of spermatogenesis

*NP* not performed

^a^Reviewed by Brisset et al., 2005 [21]

Our patient showed normal hormonal levels and normal testicular volumes, similar to previous studies [27–31]. The effect of non-obstructive azoospermia on hormone levels and testicular volume is controversial, because it has been shown that spermatogenesis disorders can result in compensatory changes in hormone levels [33]. In these cases, the seminiferous tubules may still be able to produce reproductive hormones. In addition, it has been shown that men with normal reproductive hormone levels do not necessarily have normal reproductive hormone activity [34, 35]. Further studies are needed to confirm that translocations inducing meiotic arrest do not affect hormone levels or testicular volume.

In conclusion, we describe an apparently healthy patient with a Y;autosome translocation who displayed spermatogenesis arrest leading to azoospermia and infertility. Our case highlights the importance of traditional chromosome analysis and FISH to determine the breakpoints of a reciprocal translocation. We suggest that oligospermic males with a Y;autosome translocation should pursue conception with assisted reproduction techniques such as intracytoplasmic sperm injection. Considering the risk of transmission of chromosomal abnormalities to the offspring, we also suggest genetic counseling and possibly selection of female embryos by preimplantation genetic diagnosis.
